# Antibody titers in turkeys increase after multiple booster vaccinations with an attenuated *Salmonella* live vaccine

**DOI:** 10.1186/s13104-018-3462-y

**Published:** 2018-06-08

**Authors:** Martina Hesse, Rita Weber, Gerhard Glünder

**Affiliations:** Clinic for Poultry, University of Veterinary Medicine Hannover, Bünteweg 17, Hannover, Germany

**Keywords:** *Salmonella*, Turkey, Immune response, Immunisation

## Abstract

**Objective:**

Human Salmonellosis is one of the most frequently reported foodborne zoonoses in the European Union. The most common source of human infections is the consumption of poultry products. Besides management and hygiene practices vaccination of poultry livestock is seen as one way to reduce Salmonella infections in humans. Turkey flocks in Europe are frequently infected with *Salmonella* and until recently there was no live vaccine for turkeys available. The aim of the present study was to examine the development of humoral antibodies after repeated vaccination with a bivalent live *Salmonella* vaccine containing attenuated *Salmonella* Typhimurium and *Salmonella* Enteritidis strains. Furthermore the colonization of the caecum with the vaccine strains and their spread to liver and spleen as well as the course of their fecal excretion was observed.

**Results:**

Antibody production was hardly detectable after the first vaccination but increased after booster vaccinations. Both the *Salmonella* Enteritidis and the *Salmonella* Typhimurium vaccine strain were reisolated from caecum contents and organ samples. After booster vaccinations the re-isolation rates were reduced. The shedding of the vaccine strains was most pronounced after the first vaccination.

**Electronic supplementary material:**

The online version of this article (10.1186/s13104-018-3462-y) contains supplementary material, which is available to authorized users.

## Introduction

Infections with non-host-specific *Salmonellae* are often subclinical in poultry. As a zoonotic agent however *Salmonellae* pose a serious threat to public health. The bacteria may be introduced into the food chain and cause enteritis in humans. In people at risk such as infants, small children, and the elderly, *Salmonella* infections may be serious. Therefore the primary aim of *Salmonella* control in poultry is to reduce the contamination of poultry products and subsequently the transmission to humans. At the same time very young animals poults might profit from the protection against *Salmonella* Typhimurium (ST) which may cause severe disease with great economic losses in the early stage of life.

Vaccination of livestock as a tool to reduce human *Salmonellosis* has been researched for decades now. However, not much work has been dedicated to the vaccination of turkeys [1]. Krüger et al. [2] tested a live vaccine for *Salmonella* Enteritidis (SE) in turkeys but found it unsuitable for reduction of shedding or prevention of systemic spread of virulent SE. Different types of killed vaccines have been reported to reduce shedding of SE and internal organ colonization of SE in turkeys, and to confer protection that is passed on to the progeny of vaccinated breeders (reviewed by [3]). Thain et al. [4] reported high IgG anti body titers after vaccination of turkey breeders and high levels of maternal antibodies in their offspring. Tenk et al. [5] observed low serum antibody titers after vaccination of turkeys but better performance. Overall however, it has been argued that live vaccines are better suited to stimulate cellular immunity and to confer protection in poultry [6]. For turkeys more information on the use of live vaccines is needed. Recently, two studies by our group addressed the use of a combined *Salmonella* Enteritidis (SE)- and *Salmonella* Typhimurium (ST)-life vaccine in turkey poults. After vaccination at day of hatch the ability of the vaccine to stimulate immune responses was evaluated. Several immune parameters were examined after vaccination, including the measurement of IgG serum antibody titers by ELISA until 3 weeks of age, but no increase in antibody titers in vaccinated turkey poults could be observed [7]. However, in several studies concerning *Salmonella* vaccines and other vaccines in turkeys antibody production began at three to 4 weeks of age independently from the timepoint of vaccination [8–10]. Also, only few birds were used in our previous study. The objective of the present study was therefore to determine if antibody production could be detected, if certain parameters were changed. This included that vaccinated birds were observed for a longer period of time, that a greater number of birds was used and that birds received booster vaccinations. To our knowledge antibody production after *Salmonella* vaccination has not been studied before over such a long period of time in turkeys. Additionally we examined the invasiveness of the vaccine strains and their excretion after vaccination at the 1st day of life and after the respective booster vaccinations.

## Main text

### Materials and methods

#### Experimental design, sample collection and preparation

Commercially available fattening turkeys, type BUT Big 6 (Moorgut Kartzfehn von Kameke GmbH & Co. KG, Germany) were used for the experiments. Multiple regular bacteriological control of the parent flock and serological examination of poults proved the *Salmonella* free status of the birds.

On the day of hatching, female turkey poults were randomly divided into two groups of 76 birds each. One group (Additional file 1: Table S1) was vaccinated with the bivalent SE/ST vaccine at the 1st day of life with booster vaccinations at 6, 16 and 23 weeks of age, whereas the other group remained untreated as control group. Generally on days 3, 7, 14 and 21 after each vaccination and additionally on day 5 after the first vaccination five animals were sacrificed by exsanguination after they had been stupefied by manually applied blunt force trauma. Samples from liver, spleen and caecal contents were examined for the presence of both vaccine strains. The fecal excretion of the vaccine strains was monitored by culture of cloacal swabs collected from 24 birds 1, 2, 3, 5, 7, and 14 days and then weekly after each vaccination. Blood samples from the same animals as well as from 24 unvaccinated birds of the control group were tested weekly for serum antibody titers.

#### Vaccine strains and culture

In the present study newly hatched turkey poults were immunised with a commercial live vaccine licensed for the protection of chickens, ducks and turkeys against *Salmonella* infections (“AviPro SALMONELLA VAC E+T”, Lohmann Animal Health GmbH & Co KG, Cuxhaven, Germany. It contains the attenuated *S.* Typhimurium strain ST Nal2/Rif9/Rtt and the *S.* Enteritidis strain SE Sm24/Rif12/Ssq [11]. The lyophilised vaccine was prepared to contain 1 × 10^8^ cfu of each strain per dose. The correct dosage was verified afterwards by a serial tenfold dilution in buffered peptone water and subsequent culture on a solid medium.

#### Bacteriology

Bacteriology comprised qualitative re-isolation of the vaccine strains from the caecum ingesta, liver and spleen and from cloacal swabs. Therefore organ pieces of approximately 1 g or cloacal swabs, respectively, were added to 9 mL of buffered peptone water and incubated at 38°. After 24 h culture medium was streaked with a sterile loop on agar plates supplemented with either 100 µg/mL Rifampicin and 5 µg/mL Nalidixic acid to select the ST vaccine strain or with 100 µg/mL Rifampicin and 200 µg/mL Streptomycin to select the SE vaccine strain. The identity of the *Salmonella* colonies could be further confirmed by the affiliation to different serogroups using *Salmonella* test sera (REF ORND03 and REF ORNH03 by Dade Behring, Marburg, Germany).

#### ELISA

For antibody detection, the commercially available antibody test kits Salm Gp B and Salm Gp D BioChek (item numbers CK 118 and 117, BioChek, Reeuwijk, the Netherlands) were used in accordance with the manufacturer’s instructions. The former detects antibodies against LPS-antigen of the *Salmonella* serogroup B (including serovar *Salmonella* Typhimurium) according to the Kauffmann-White scheme and the latter detects antibodies against antigens of serogoup D (including *Salmonella* Enteritidis).

### Results and discussion

#### Re-isolation of Salmonella vaccine strains from caecal ingesta, liver, spleen and cloacal swabs after repeated vaccinations of turkeys (Fig. 1)

Three and 5 days after the first vaccination the SE vaccine strain and on days 3, 5 and 7 the ST strain could be re-isolated from the caecal ingesta of all sacrificed birds. Afterwards re-isolation rates dropped but the ST-strain could still be detected until day 21 post infection in one bird. At day 14 and 21 post vaccination the SE-strain was not detected in caecum ingesta anymore. The re-isolation rates from liver and spleen were lower than those from caecum. The ST-vaccine strain was re-isolated from day 3 after vaccination from liver and from day 5 from spleen and reached a re-isolation rate of 60% at day 5 in the liver and at day 7 in the spleen. After day 14 post vaccination the re-isolation rate began to drop. The strain was not detected in the liver at day 21 post vaccination. The SE-strain could not be re-isolated from the liver and only from the spleen of one animal at day 14 after vaccination.

**Fig. 1:**
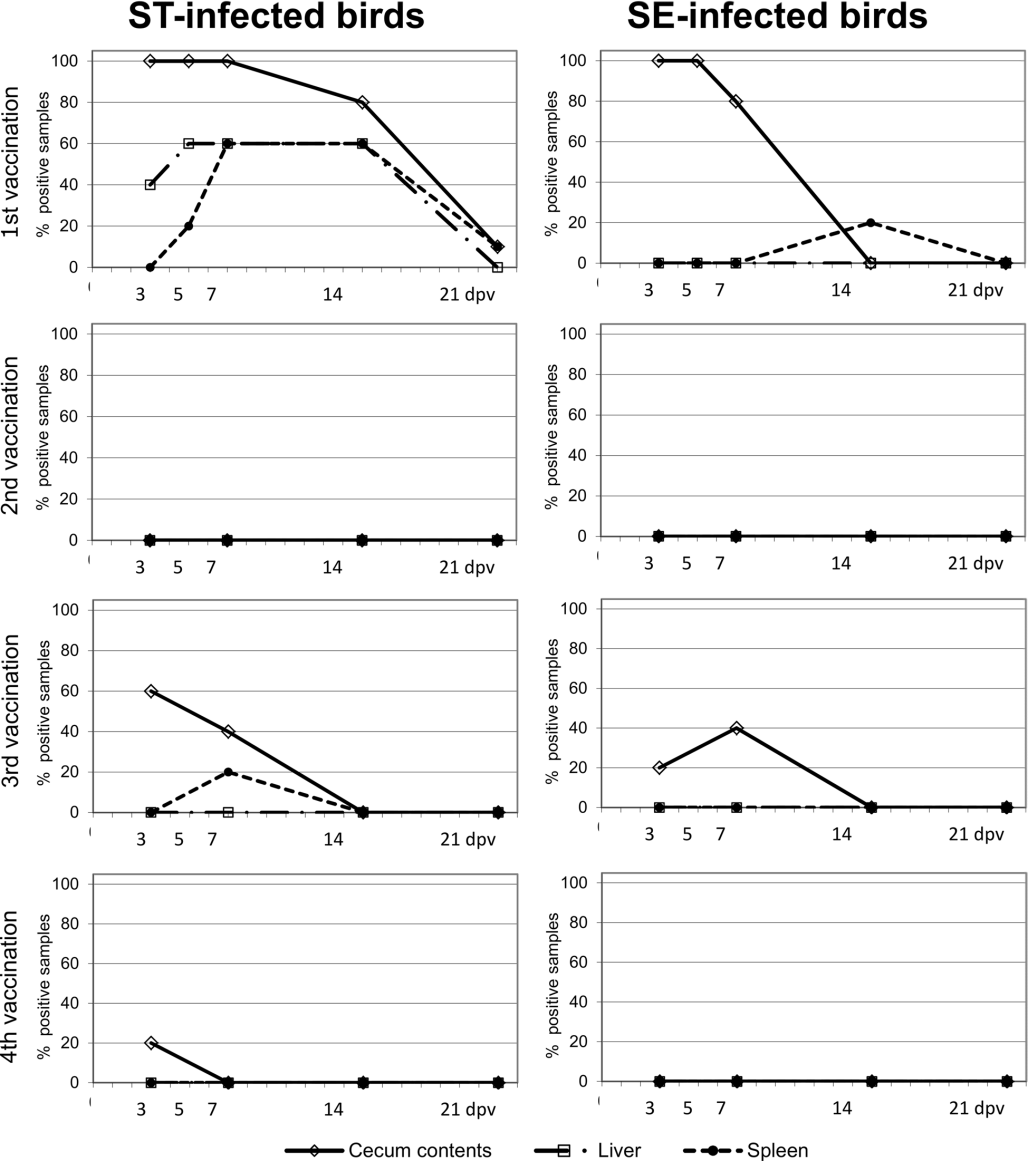
Re-isolataion of the vaccine strains from caecum ingesta, liver and spleen. At each timepoint 5 birds per group were tested

After the second vaccination at an age of 6 weeks (1st booster vaccination) neither of the vaccine strains could be re-isolated from any of the samples. After the third vaccination at 16 weeks (2nd booster vaccination), re-isolation rates from all sample types were lower compared to the first vaccination and even lower still after the fourth vaccination (3rd booster vaccination). After the third vaccination both vaccine strains could be detected in cecal contents, the ST strain at day 3 and 7 from three and two cecal samples, respectively, the SE strain only at day 7 pv from two samples. Only the ST strain could be re-isolated from one organ sample. After the fourth vaccination at an age of 23 weeks (3rd booster vaccination) the ST-strain could be re-isolated only from the caecum ingesta of one bird at day three post vaccination whereas the SE-vaccine strain could not be re-isolated at all. The fact that the ST vaccine strain colonized the cecum for a longer time and showed more often invasive behavior might reflect the greater virulence of wildtype ST strains for turkeys [3].

**Fig. 2:**
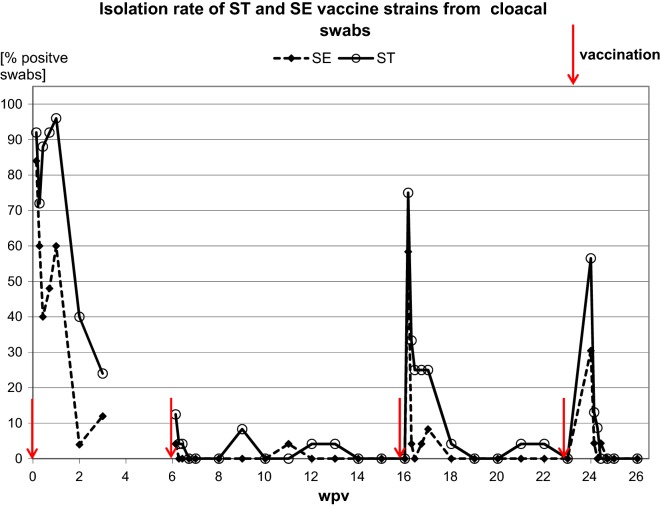
Re-isolation of the vaccine strains from cloacal swabs. At each timepoint 24 birds were tested

The ability of the *Salmonella* vaccine to colonize the caecum and to be invasive is associated with its immunogenicity and therefore with its efficacy. Methner et al. [12] observed that the number of colony forming units of a *Salmonella* strain present in the caecum is associated with the antibody-response and the protectiveness against infection with virulent strains, although the antibody response might not be the cause of protection. Moreover, Berndt et al. [13] observed that a high immunogenicity of a *Salmonella* vaccine is dependent on a certain invasiveness of the *Salmonella* strains. The vaccine strains used in the present study colonized the caecum but spread systemically only to a minor extend. However, when administered to chickens, the same vaccine could not be detected in any sample (caecum, organ or cloacal swab) but was nevertheless protective in challenge trials [11]. It has also been shown to confer protection against *Salmonella* infection in turkeys [7]. After the first vaccination the ST-strain could be isolated from around 90% of the cloacal swabs (Fig. 2) in the 1st days post vaccination. This rate dropped to 24% at 3 weeks. After the third and the fourth vaccination the course of shedding was similar, but re-isolation rates were lower after the third vaccination compared to the first and again lower after the fourth vaccination. Similar to re-isolation rates from organ samples the re-isolation rates from cloacal swabs were lower for the SE-strain compared to the ST-strain after each vaccination. The course of shedding after the second vaccination deviates from this pattern. Shedding began with 12.5% at day one post vaccination for both strains. At day 5 post vaccination the ST-strain could not be isolated from any bird but again at day 21 post vaccination from two birds. The SE-strain could not be found afterwards.

Overall the re-isolation from cloacal swabs was lowest after the second vaccination, possibly due to the short 6-week interval after the previous first vaccination. If re-isolation rates after the second vaccination are regarded as some random deviation, one could argue that older birds were better able to prevent caecal colonization and systemic spread of the vaccine strains than younger birds. The ability of turkey to clear *Salmonella* infection has been shown to be age dependent due to the immune system of turkey not being fully mature at hatch [14, 15].

It would also be possible that with each booster vaccination the immune protection against *Salmonella* infection increased [16, 17]. The memory immune response might have impeded the systemic spread and even the caecum colonization of the vaccine strains.

It is interesting that the re-isolation rates of the SE—strain were consistently lower than the ones of the ST-strain. It is known that some *Salmonella* strains inhibit the infection with other *Salmonella* strains [18]. However, the vaccine strains which were examined in the present study did not show an inhibitory effect on each other in chickens.

#### Antibody-titers in serum (Fig. 3)

After the first vaccination antibody titers and remained beneath the cutoff of the test (0.5), which corresponds with prior research by our group and others [7–9]. This can be explained with the immaturity of the turkey immune system at this age [14, 19, 20]. After the second vaccination antibody titers increased for 6 weeks before titers dropped below the cutoff again. After the third vaccination antibody titers peaked after 2 weeks and reached higher values but dropped faster as well. Five weeks later the titers had returned to the values of the time before vaccination. The antibody response may have been stronger due to the increased age of the birds [20, 21]. After the fourth vaccination the antibody response was similar to the one after the third vaccination. A low antibody response like after the first vaccination does not preclude the efficacy of the vaccine against challenge infections [22]. In chickens antibody production is not always correlated with protection. [23–25] and it has been suggested that the antibody response is not the cause of protection [24].

**Fig. 3:**
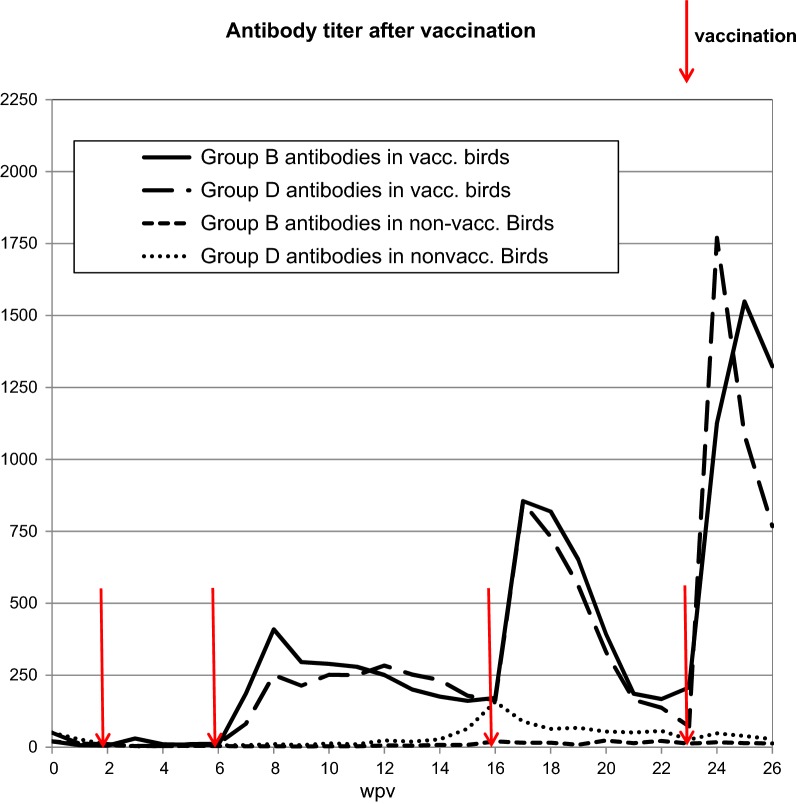
Antibody titers after vaccinations. At each timepoint 24 birds per group were tested

### Conclusion

The immunogenicity of the vaccine was demonstrated by the observed antibody response. Each booster stimulated the antibody production. The colonization with the vaccine strains was impaired in older birds that had been vaccinated more often.

## Limitations

Standard deviations for antibody titers were very high, which makes the interpretation of the data difficult.

## Additional file

**Additional file 1: Table S1.** Experimental design. Age of birds at vaccination and time points of sampling vaccination in days after vaccination. Microbiological samples were collected from vaccinated birds, serum samples were collected from vaccinated bird and an equal number of unvaccinated birds.

## Abbreviations

SE: *Salmonella* Enteritidis
ST: *Salmonella* Typhimurium
