# Supplementary material for: Antibody titers in turkeys increase after multiple booster vaccinations with an attenuated Salmonella live vaccine
Source: BMC Res Notes. 2018 Jun 8;11:367. doi: 10.1186/s13104-018-3462-y (PMC5994122; doi:10.1186/s13104-018-3462-y)
Supplement: Supplementary file 1 — Additional file 1: Table S1. Experimental design. Age of birds at vaccination and time points of sampling vaccination in days after vaccination. Microbiological samples were collected from vaccinated birds, serum samples were collected from vaccinated bird and an equal number of unvaccinated birds. [file 13104_2018_3462_MOESM1_ESM.docx]

**Table S1: Experimental design**

| **No. of vaccination** | **Age at vaccination** | **Microbiological samples** | | **Serum samples** |
| --- | --- | --- | --- | --- |
|  |  | **liver, spleen, caeca**  **5 turkeys** | **Cloacal swabs**  **24 turkeys** | **24 turkeys** |
|  |  | days post vaccination | days post vaccination |  |
| 1^st^ | 1 day | 3, 5, 7, 14, 21 | 1, 2, 3, 5, 7, 14, 21 | weekly during the entire experimen-tal period of 26 weeks |
| 2^nd^ | 6 weeks | 3, 7, 14, 21 |  |  |
| 3^rd^ | 16 weeks |  |  |  |
| 4^th^ | 23 weeks |  |  |  |
